# Reporting guideline for priority setting of health research (REPRISE)

**DOI:** 10.1186/s12874-019-0889-3

**Published:** 2019-12-28

**Authors:** Allison Tong, Anneliese Synnot, Sally Crowe, Sophie Hill, Andrea Matus, Nicole Scholes-Robertson, Sandy Oliver, Katherine Cowan, Mona Nasser, Soumyadeep Bhaumik, Talia Gutman, Amanda Baumgart, Jonathan C. Craig

**Affiliations:** 1 Sydney School of Public Health, University of Sydney, Sydney, NSW Australia; 2 Centre for Kidney Research, The Children’s Hospital at Westmead, Westmead NSW, Sydney, 2145 Australia; 3 Centre for Health Communication and Participation, School of Psychology and Public Health, La Trobe University, Melbourne, Victoria Australia; 4 Cochrane Australia, School of Public Health and Preventive Medicine, Monash University, Melbourne, Victoria Australia; 5 Crowe Associates Ltd, Oxon, UK; 6 Institute of Education, University College London, London, UK; 7 Faculty of Humanities, University of Johannesburg, Johannesburg, South Africa; 8 Katherine Cowan Consulting Ltd, East Sussex, UK; 9 Peninsula Dental School, University of Plymouth, Plymouth, UK; 10 The George Institute for Global Health, New Delhi, India; 11 College of Medicine and Public Health, Flinders University, Adelaide, South Australia Australia

**Keywords:** Priority-setting, Reporting, Transparency, Patient involvement

## Abstract

**Background:**

Research priority setting with stakeholders can help direct the limited resources for health research toward priority areas of need. Ensuring transparency of the priority setting process can strengthen legitimacy and credibility for influencing the research agenda. This study aims to develop a reporting guideline for priority setting of health research.

**Methods:**

We searched electronic databases and relevant websites for sources (frameworks, guidelines, or models for conducting, appraising, reporting or evaluating health research priority setting, and reviews (including systematic reviews)), and primary studies of research priority setting to July 2019. We inductively developed a list of reporting items and piloted the preliminary guideline with a diverse range of 30 priority setting studies from the records retrieved.

**Results:**

From 21,556 records, we included 26 sources for the candidate REPRISE framework and 455 primary research studies. The REporting guideline for PRIority SEtting of health research (REPRISE) has 31 reporting items that cover 10 domains: context and scope, governance and team, framework for priority setting, stakeholders/participants, identification and collection of priorities, prioritization of research topics, output, evaluation and feedback, translation and implementation, and funding and conflict of interest. Each reporting item includes a descriptor and examples.

**Conclusions:**

The REPRISE guideline can facilitate comprehensive reporting of studies of research priority setting. Improved transparency in research priority setting may strengthen the acceptability and implementation of the research priorities identified, so that efforts and funding are invested in generating evidence that is of importance to all stakeholders.

**Trial registration:**

Not applicable.

## Background

Historically, the health research agenda has been largely investigator-driven with limited input from other stakeholders including patients, caregivers and the community [1, 2]. Given the evident mismatch between the research interests of patients and researchers, investment into health research may be misdirected to areas of low priority or fail to address important needs of relevant stakeholders [1, 3–8]. For example, an analysis of 14 research priority setting partnerships involving patients with different medical conditions found that pharmacological interventions were prioritized only in 18% of the total priorities but 58% of the clinical trials in those fields evaluated pharmacological interventions [4].

Globally, there have been calls for research priority setting with stakeholders to be done at all levels of health systems, jurisdictions, and health areas [9–12]. There is no consensus on the definition of research priority setting but most definitions refer to a range of activities that involve identifying, prioritizing, and achieving consensus on the research areas or questions of importance to stakeholders [13, 14]. The past two decades have seen increasing efforts to develop better ways to engage all relevant stakeholders, particularly patients, in setting priorities for research across different health disciplines and populations [15–17]. Involving stakeholders in an explicit manner in research priority setting can help to: 1) ensure that funding decisions and research meet critical evidence gaps to inform decision making; 2) facilitate shared responsibility and accountability in implementing the research agenda; 3) improve the relevance and legitimacy of research; and 4) ultimately achieve better health outcomes [12, 17].

A diverse range of methods are used to prioritize research given the different healthcare contexts, populations, environments and resources available in which the priority setting is undertaken [14]. The process of research priority setting can be complex, political and value-laden. It can also be challenging to identify, address and integrate the different perspectives and values held by diverse stakeholders. While there is no consensus on what constitutes “successful” research priority setting, it has been advocated that processes must be fair, legitimate, informed by credible evidence, involve a broad spectrum of stakeholders, and be transparent [12, 13, 18–20].

However, reviews of published research priority setting exercises have consistently demonstrated a lack of transparency because of suboptimal reporting [17, 21–26]. A systematic review of research priority setting in childhood chronic disease, in which most studies were conducted in the UK, US, and Australia, found that methods for collecting and prioritizing research topics were reported in only 50 (60%) of the 84 studies included [23]. Another review of research priority setting exercises in Zambia reported that details about the process and the stakeholders involved were omitted in the majority of studies [22], and similarly, a review of studies in the Islamic Republic of Iran revealed that 22 (61%) of the 36 priority setting studies did not report methods and only listed the research priorities [21]. Inadequate description of the stakeholders and the methods makes it difficult to assess the validity of research priorities identified, and limits the ability to aggregate, analyze or compare research priorities that have been established [27].

Frameworks and guidelines are available for conducting and evaluating research priority setting, which mainly focus on criteria related to the process rather than the outcomes and impact of priority setting. There are no published guidelines for reporting priority setting for health research [5, 13, 18, 27–29]. Ensuring the transparency of the process for research priority setting can strengthen legitimacy and credibility to support implementation and maximise impact. A reporting checklist for research priority setting may facilitate more consistent and comprehensive reporting and enable researchers and end-users to better understand the processes taken in developing research priorities. The aim of this paper is to introduce the reporting guideline for priority setting of health research (REPRISE), describe its development and provide a rationale for the items included.

## Methods

### REPRISE development

We used the Enhancing the Quality and Transparency of Health Research (EQUATOR) toolkit [30], for developing the REPRISE Guideline and reported our approach based on the “Guidance for developers of health research reporting guidelines” where possible [31]. We have also registered REPRISE with the EQUATOR Network.

#### Purpose and context

The purpose of REPRISE is to facilitate comprehensive and transparent reporting of health research priority setting exercises, in which there is direct involvement of stakeholders setting research priorities. The REPRISE guideline is flexible so that it may be used for a range of approaches. The scope of REPRISE does not cover approaches without direct involvement of stakeholders such as documentary analysis (e.g. evidence mapping), and econometrics methods (e.g. value of information). REPRISE is not intended for use to appraise the quality of priority setting studies, establish or evaluate criteria for research priorities (e.g. evidence gaps, prevalence of disease, economic considerations), and does not recommend a preferred approach.

#### Identify the need for a guideline

Systematic reviews have consistently shown the reporting of the process of research priority setting with stakeholders is highly variable and limited with many details omitted [21, 23, 25, 26, 32]. The need for a reporting guideline has also been identified through our workshops and forums at national and international meetings (e.g. Cochrane Colloquia and Symposia [12, 33–35]; James Lind Alliance [5]), and through our collective experiences of conducting, publishing, reviewing, and using research priority setting studies.

We conducted a comprehensive search for frameworks, guidelines or models for conducting, appraising, reporting and evaluating health research priority setting, reviews (including systematic reviews) of research priority setting studies, and primary research priority setting studies. We searched electronic databases including MEDLINE, Embase, CINAHL, PsycINFO from inception to 23rd July 2019 using sensitive search strategies provided in Additional file 1. We used Medical Subject Heading (MeSH) terms and text words for research priorities and combined this with terms related to reporting, conduct and evaluation. We also searched Google Scholar, relevant organizational websites (e.g. WHO, EQUATOR, Cochrane, James Lind Alliance and PCORI), and reference lists of articles. The search results are shown in Additional file 2. From the 21,556 records retrieved, we identified 13 frameworks or guidelines for conducting or evaluating research priority guidelines [5, 13, 18–20, 28, 36–42] (none designed for reporting research priority setting), and 13 reviews of research priority setting [15–17, 21–27, 32, 43, 44], of which four were systematic reviews [23–26]. (Additional file 3) We also identified 455 primary research priority setting studies.

#### Generating reporting items for the candidate checklist

We extracted items related to the process of priority-setting from the frameworks and systematic reviews included (the sources are listed in Additional file 2). We translated these into reporting items for the candidate REPRISE reporting guidelines by grouping similar items and rephrasing the statements as a reporting item. We inductively developed the initial list of reporting items. This was reviewed by two other investigators (AB, AM) to ensure all relevant items were included in the list. The reporting items were compiled into 10 domains: context and scope, governance and team, framework for priority setting, stakeholders/participants, identification and collection of priorities, prioritization of research topics, output, evaluation and feedback, translation and implementation, and funding and conflict of interest. (Additional file 4) We imported all sources (frameworks and systematic reviews) into HyperRESEARCH software for coding textual data, and AT conducted line-by-line coding of each source to the initial items. We generated a report of each reporting item and the corresponding coded text (content). We developed descriptors and examples based on the content of the sources and input from all the investigators. The sources that contributed to each reporting item, and examples of the original extracted items are shown in Additional file 5.

#### Pilot testing the checklist

The preliminary REPRISE guideline was presented at the Australasian Cochrane Symposium, in which participants used the guideline to assess the reporting of a research priority exercise, and provided feedback on the guideline [34]. We subsequently used the REPRISE guideline in two systematic reviews of research priority setting studies in childhood chronic conditions [23] and organ transplantation [26]. We also applied the preliminary guideline to report a research priority setting exercise in health communication and participation [45].

In the final stage, we piloted the guideline with research priority setting studies. We used a purposive sampling strategy to select 30 priority setting exercises from the total of 455 studies retrieved from the search to ensure a diverse range of health topics, regions, stakeholders involved, framework or methods used, and type of output. Using a standardized data extraction template with the reporting items from the REPRISE guideline, the investigators (two per study) independently assessed if the study reported on each item (yes/no), added comments, and suggested new reporting items that were not yet captured. The results are provided in Additional file 6. After completion, the investigators discussed the relevance and applicability of the items, clarity of the items, comprehensiveness of the descriptor and examples, and any new reporting items proposed. These were integrated into the final reporting guideline, which was reviewed and approved by all investigators.

### REPRISE framework: content and rationale

The REPRISE guideline includes ten domains and a total of 31 reporting items with a descriptor and examples provided. (Table 1) The principles, rationale, and explanation for the domains are detailed below, which are based on the synthesis of sources listed in Additional file 3, priority setting studies, and discussion among the investigators.

**Table 1:** Reporting guideline for health research priority setting with stakeholders (REPRISE)

No	Item	Descriptor and/or examples
A	Context and scope	
1	Define geographical scope	Global, regional, national, city, local area, institutional/organizational level, health service
2	Define health area, field, focus	Disease or condition specific, interventions, healthcare delivery, health system
3	Define the intended beneficiaries	This may include the general population or a specific population based on demographic (age, gender), clinical (disease, condition), or other characteristics who may benefit from the research
4	Define the target audience of the priorities	Policy makers, funders, researchers, industry or others who have the potential to implement the priorities identified
5	Identify the research area	Public health, health services research, clinical research, basic science
6	Identify the type of research questions	Etiology, diagnosis, prevention, treatment (interventions), prognosis, health services, psychosocial, behavioral and social science, economic evaluation, implementation; this may not be pre-defined
7	Define the time frame	Interim, short-term, long-term priorities, plans to revise and update
B	Governance and team	
8	Describe the selection and structure of the leadership and management team	Those responsible for initiating, developing, and guiding the process for priority setting, and examples of structures include; Steering Committee, Advisory Group, Technical Experts
9	Describe the characteristics of the team	Stakeholder group or role, institutional affiliations, country or region, demographics (e.g. age sex), discipline, experience, expertise
10	Describe any training or experience relevant to conducting priority setting	Consultants or advisors, members with experience or skills relevant to the conducting priority-setting e.g. qualitative methods, surveys, facilitation
C	Framework for priority setting	
11	State the framework used (if any)	James Lind Alliance, COHRED, CHNRI, Dialogue Model, no framework (general research priority setting)
D	Stakeholders or participants	
12	Define the inclusion criteria for stakeholders involved in priority-setting	Patients, caregivers, general community, health professionals, researchers, policy makers, non-governmental organizations, government, industry; specific groups including vulnerable and marginalized populations
13	State the strategy or method for identifying and engaging stakeholders	Partnership with organizations, social media, recruitment through hospitals
14	Indicate the number of participants and/or organizations involved	Number of individuals and organizations, include number by stakeholder group
15	Describe the characteristics of stakeholders	Stakeholder group, demographic characteristics, areas of interest and expertise, discipline, affiliations
16	State if reimbursement for participation was provided	Cash, vouchers, certificates, acknowledgement; what purpose e.g. travel, accommodation, honorarium
E	Identification and collection of research priorities	
17	Describe methods for collecting initial priorities	Methods e.g. Delphi survey, surveys, nominal group technique, interviews, focus groups, meetings, workshops; prioritization e.g. voting, ranking; mode e.g. face-to-face, online; may be informed by evidence e.g. systematic reviews, reviews of guidelines/other documents, health technology assessment
18	Describe methods for collating and categorizing priorities	Taxonomy or other framework used to organize, summarise, and aggregate topics or questions
19	Describe methods and reasons for modifying (removing, adding, reframing) priorities	Based on scope, clarity, definition, duplication, other criteria
20	Describe methods for refining or translating priorities into research topics or questions	Reviewed by Steering Committee or project team
21	Describe methods for checking whether research questions or topics have been answered	Systematic reviews, evidence mapping, consultation with experts
22	Describe number of research questions or topics	Number of priorities at each stage of the process
F	Prioritization of research topics/questions	
23	Describe methods and criteria for prioritizing research topics or questions	Methods e.g. Delphi survey, surveys, nominal group technique, interviews, focus groups, meetings, workshops; Prioritization e.g. voting, ranking; Mode e.g. face-to-face, online; Criteria e.g. need, feasibility, novelty, equity
24	State the method or threshold for excluding research topics/questions	Thresholds for ranking scores, proportions, votes; other criteria
G	Output	
25	State the approach to formulating the research priorities	Area, topic, questions, PICO (population, intervention, comparator, outcome)
H	Evaluation and feedback	
26	Describe how the process of prioritization was evaluated	Survey, workshop
27	Describe how priorities were fed back to stakeholders and/or to the public; and how feedback (if received) was addressed and integrated	Public meetings or workshop, newsletters, website, email, online presentations
I	Implementation	
28	Outline the strategy or action plans for implementing priorities	Communication with target audience, via policies and funding
29	Describe plans, strategies, or suggestions to evaluate impact	Integration in decision-making, funding allocation, review of relevant documents
J	Funding and conflict of interest	
30	State sources of funding	Name sources of funding for the priority-setting exercise; if relevant include the budget and/or cost
31	Declare any conflicts or competing interests	State any conflicts of interest that may be at an individual level and/or at a contextual level (e.g. political issues, controversies) that may affect the process, output or implementation.

#### Context and scope (items 1–7)

Establishing the context and scope is recommended as these “underpin the process of research priority setting,” [13] including the selection of relevant stakeholders and methods used. In terms of geographical scope, priority setting may be done at an institutional, local, national, or international level [13, 19, 21, 39–41]; recognizing that each will have its own “sense of mandate, capacity, culture, and resources.” [39] Research priorities may address a specific condition, disease or risk factor (e.g. cancer, mental health), population (e.g. elderly, adolescents), health system, research design, or interventions (e.g. vaccination) [5, 13, 19, 21, 24, 27, 37, 38, 40, 41, 44]. These can be decided upon based on the evidence [5, 38] and initial deliberations with stakeholders [38].

The intended beneficiaries [13] may include patients, caregivers, or the general community who could benefit from the priority setting exercise, and the target audience are those who have the potential to implement or fund the research priorities identified [13, 17, 39–41]. The focus, content and type of research to be considered can determine the scope of the priority setting exercise. In terms of the broad research areas, these generally span public health, health services, clinical research and basic science [24, 37, 41, 44]. The types of research questions that may be included can range from etiology, diagnosis, prognosis, treatment, to behavioural; and social science, economic evaluation and implementation [23, 24, 27, 39]. It is not necessary for the type of research questions to be determined a priori.

Providing an estimated time frame that the priorities are expected to be valid or relevant may be relevant. This is because research priorities may evolve due to the development of new technology or interventions, emerging evidence, or changes to the health system or socio-political contexts [13, 17, 19, 37–39, 41, 42]. If there are plans to update the priority setting or to monitor the priorities for the need to update, these could be described. There has been suggestion of 3–5 year cycles of prioritization if the priority setting exercise is to be repeated [37, 38].

#### Governance and team (items 8–10)

It has been argued that priority setting requires “credible” [39] leadership to support acceptability and uptake. This may require leaders who are trusted by stakeholders and who have the necessary expertise, knowledge, decision-making skills, and ability and deliver the project. The leadership and management team is usually responsible for overseeing, developing and implementing the process for priority setting [5, 13, 19]. The leadership group may take the form of, for example, an Executive Committee, Advisory Group, Technical Expert Group [13]. Members of the leadership team would generally be expected to contribute broad and relevant collective insights, harness their networks for engagement and partnership; and include a diversity of members to offer legitimacy to wider stakeholder networks (e.g. patients, caregivers, researchers, policy makers, clinicians, representatives from other non-government or government organizations) [5, 21], and those with technical expertise [5]. The membership and selection of stakeholders may need to take into consideration the need for equity [12, 36]. Also, it has been suggested that the involvement of individuals or organizations with experience in priority setting and relevant research skills can ensure a “high quality process.” [5, 13, 40] With regard to facilitators, neutrality and facilitations skills may be important to elicit input from diverse and mixed stakeholders [5, 36].

#### Framework for priority setting (item 11)

Some priority setting studies use or adapt frameworks to guide the process [17, 22, 23, 25, 44]. Common frameworks include the James Lind Alliance [5], Council on Health Research for Development Essential National Health Research (COHRED/ENHR) [19], Essential National Health Research (EHNR), Child Health and Nutrition Research Initiative (CHMRI) [41], and the Dialogue Model [36]. A summary of these frameworks is provided in Table 2. Some priority setting exercises may develop and use a different approach, and not necessarily follow an existing or established framework.

**Table 2:** Summary of frameworks for conducting health research priority setting

Framework	Year	Organization^a^	Country^a^	Principles/values/ characteristics	Stakeholders	Scope	Outline of process	Output
James Lind Alliance (JLA) [5]	2004	National Institute for Health Research (NIHR)	UK	Partnership	Patients, caregivers, clinicians	Diagnosis, intervention, care and support	Gather priorities (survey) Process and verify Conduct Interim priority setting (survey) Conduct final priority setting (workshops using nominal group technique)	Top 10 research questions for funders
Council on Health Research for Development Essential National Health Research (COHRED/ENHR) [39]	2000	Council on Health Research for Development	International	Inclusivity, involvement of a broad range of stakeholders, multidisciplinary and cross-sectorial, partnership, participatory and transparent, systematic analysis of health needs, societal and professional expectations	Researchers, decision-makers, health service providers, communities	–	Establish criteria Identify research areas (brainstorming, voting, nominal group technique, roundtable etc) Score against criteria (survey)	–
Child Health and Nutrition Research Initiative (CHNRI) [41, 46]	2007	Global Forum for Health Research	International	Systematic, fair, transparent	Investors in health research, researchers, general public	Fundamental, translation, implementation	Discuss criteria Select useful and important criteria Score against criteria (survey) Elicit stakeholder input (reference group) Adjust scores with stakeholder input	–
Dialogue Model [36]	2007	VU University	The Netherlands	Participatory, respect for experiential knowledge, dialogue between different stakeholders, emergent and flexible design	Patients, researchers, health professionals		Explore (informal discussion) Consult (separate stakeholder consultations, focus groups, interviews, other methods) Prioritize (survey, focus group, Delphi technique) Integrate (meeting)	–

^a^Refers to developers

#### Stakeholders or participants (items 12–16)

Stakeholder involvement in priority setting can vary across the priority setting exercises. In some cases, they are involved in all key stages of the process and in others, they are consulted in specific steps and existing data or documents are used instead of consultation. Relevant stakeholders whose “values and interests should be respected in setting health research priorities” [41] can include patients, caregivers, clinicians, policy makers, representatives from non-governmental organizations [5, 18, 21, 39, 44]; and diverse groups, for example based on demographic or clinical characteristics, may need to be included in research priority setting [5, 18, 20, 36, 37, 39, 40, 42]. It has been emphasized that patients/caregivers (and if relevant the public) need to be directly involved in the priority setting process [5, 18, 38–40, 42], as they have direct experience of the health condition or context and often have different priorities to researchers and clinicians. There is also recognition of the need to involve individuals from vulnerable or marginalized groups, particularly in equity-focused research priority setting exercises [5, 12, 21, 36, 39].

Multiple strategies may be used to engage stakeholders in the priority setting process, and this is namely through partnership with relevant stakeholder organizations [5, 39]. The number and characteristics of the participants involved enables assessment of the degree of inclusivity, diversity and equity [5, 13, 39] in priority setting processes. The characteristics to specify may include role and expertise, discipline, organizational affiliations, demographics (e.g. age, sex, socio-economics status, ethnicity), and clinical factors [13, 21–23, 36, 42, 44]. Support for patients/caregivers involved in priority setting may include reimbursement for travel, arranging care for dependents, and time [5]. This may indicate to readers the degree to which the team was able to ensure inclusivity across the different groups. Of note, there is recognition that attention must be given to power dynamics, otherwise the engagement of disadvantaged and marginalized groups may lead to “presence without voice and voice without influence.” [2] Therefore, it may be relevant to acknowledge and discuss how hierarchies and “asymmetries between stakeholders” [36] are addressed to maximize constructive and balanced interaction. For example, some groups, such as patients, may require additional time, training, resources, or other strategies to be able to engage; to have the opportunity to contribute meaningfully [36].

#### Identification and collection of research priorities (items 17–22)

Different methods and approaches are available for collecting and selecting initial research priorities from stakeholders and developing the first list of priorities. This can be one or a combination of methods including interviews, focus groups, workshops, and surveys; and consensus methods (e.g. Delphi survey, nominal group technique); and these may be conducted through various modes such as face-to-face or online [5, 13, 23, 25–28, 37–39]. Documents such as systematic reviews, technical data, and other relevant reports may be used to identify the initial list of priorities [5, 13, 18, 21, 24, 39, 40]. In some priority setting exercises, the initial list of research priorities is derived from literature or existing data rather than consultation or engagement of stakeholders [47–49].

If a wide range of different initial research priorities are submitted or identified, it can be challenging to manage and synthesise to capture the diversity of views in a concise manner, whilst also retaining the context and nuances of the submissions. They may need to be organized, usually by collating and categorizing them into themes, topics or other relevant taxonomy [5, 22, 36, 38, 39, 41]; and by removing those that are “out-of-scope,” [5] or duplicative [39]. They may then be translated into “indicative, researchable questions” [5] and edited for clarity [21, 27, 35, 36, 38]. Some priority setting exercises conduct cross checking of the priorities against the evidence (i.e. systematic reviews [5]) and evidence mapping [48, 50–52].

The number of research priorities identified at each stage vary widely [24]. Generally, 10 to 20 questions/topics are included in the final set of priorities [5, 23–26, 38, 40].

#### Prioritization of research topics/questions (items 23–24)

Prioritization techniques can include scoring, ranking, voting, and ordering, and these are usually embedded in similar methods and modes used for collecting priorities as outlined in Section E. Some frameworks and priority setting exercises use explicit criteria to prioritize questions [13, 21, 22, 38, 39]. Examples include condition-related criteria (burden of disease, variation in care and outcome, evidence gaps), and research-related criteria (resources required, likelihood of success and impact) [37, 42]. The CHNRI method proposes criteria including: answerability, attractiveness (likely to be published in high-impact journals), novelty, potential for translation, effectiveness (likely to identify better interventions), affordability, deliverability, sustainability, public opinion (acceptability to the general public), equity (leads to interventions that will be accessible to marginalized or vulnerable populations), and cost and feasibility [27, 41]. Using specific criteria can facilitate a deliberative and rational process, particularly when there is limited information [21]. It may be relevant to report the processes for selecting, defining and changing the criteria. Of note, the use of criteria can add complexity to the process, and strategies may be needed to avoid inadvertent exclusion of other stakeholder values that influence prioritization. Whilst assigning scores based on such criteria may be rational, there are concerns that it may give a false sense of objectivity. The method for excluding priorities at this stage i.e. based on a quantified threshold or other criteria should be provided. Any processes to appeal or challenge the results may be specified.

#### Output (item 25)

The output should be “clear and of value to the research community.” [5] The final priorities generated can range from having a specific structure i.e. the Population, Intervention, Comparator, Outcome (PICO) format [5, 37], to broader outputs such as topics or themes/areas [38]. It is possible that components of PICO are not specified in original submission of priorities, or that it cannot be applied to some types of research questions [5]. In some circumstances, attempting to produce very technical research questions can potentially place non-researchers, who may include community members, patients, caregivers, at a disadvantage, as they may feel unable to articulate or consider the specific technical components. Also, consideration may need to be given to ensure that the contextual data and values around the questions are not missed. Some priority setting exercises seek to identify broader themes or areas and translate these into research questions after prioritization.

#### Evaluation and feedback (items 26–27)

While there is no “gold standard” [37] approach for evaluating the process of research priority setting, process evaluation can provide information about the acceptability, “reliability and usefulness” [37] of the process and results [5, 13, 37]. Stakeholder satisfaction with the process in terms of being able to engage and express opinions, and whether the priorities are considered meaningful and valid may be evaluated [13, 18]. Participants and stakeholders could have an opportunity to review and provide feedback on the prioritized questions [22, 36, 39]; and having “revision or appeal” [18] mechanisms available to identify and address disagreements in a constructive manner [18] have been suggested.

#### Implementation (items 28–29)

Strategies to implement the research priorities could involve informing and garnering support from government, policy makers, and funding agencies to allocate funding and resources toward the priorities identified [13, 19, 22, 39], and working with researchers to develop proposals [5, 19, 39]. Assessing the impact of research priority setting is challenging but needs to be considered [5, 22]. This may include the impact on decision-making, allocation of funding and resources, and research output [18, 19, 38].

#### Funding and conflict of interest (items 30–31)

There are different sources of funding that can affect a priority setting process. The funding and resources used to conduct the priority setting exercise and support the stakeholders (directly or indirectly) and whether the priority setting exercise is connected to a funding source to support the identified research priorities. Reporting the sources of funding and support is usually required. The resources required for research priority setting will depend on the size, scope, timeline, methods used, and personnel required [5, 19], and providing information about the budget may be useful for others who are planning on conducting research priority setting. It is recommended that any relevant disclosures be stated for transparency, to allow assessment of potential political or commercial influences or undue bias [5, 13, 24]. For example, pharmaceutical companies may have close ties with patient organisations and clinicians, and the potential influence this may have on the priority setting process would need to be addressed explicitly. This may be declared at an individual level, or at a process or contextual level, for example, providing a narrative of any political issues, conflict or controversies that may affect the process, output or implementation of the priority setting exercise [20].

## Discussion

The REPRISE Guideline is intended to facilitate transparent and comprehensive reporting of research priority setting studies that involve stakeholders. The guideline has 31 reporting items that cover 10 domains: context and scope, governance and team, framework for priority setting, stakeholders/participants, identification and collection of research priorities, prioritization of research topics, output, evaluation and feedback, translation and implementation, and funding and conflict of interest. The REPRISE guideline is flexible without being unduly prescriptive because different approaches for health research priority setting are necessary to ensure they are contextually appropriate, respect the underpinning values and criteria, and are feasible based upon resources available. By piloting the guideline with a broad selection of research priority setting studies, we have demonstrated the feasibility, acceptability and relevance of the REPRISE reporting guidelines. We emphasise that REPRISE is not designed for making judgements about the quality of the conduct in research priority setting studies.

The REPRISE guideline may be used as a roadmap for reporting research priority setting studies, or to assess reporting of research priority setting studies as has been done in systematic reviews [25, 26]. REPRISE is focussed on the reporting of process or conduct and does not address in detail the values and criteria for establishing priorities, though these may be described in reporting the process of prioritizing research topics/questions. We did not conduct a Delphi survey, which has been used in other reporting guidelines to prioritize and achieve consensus on what reporting items should be included [30, 31]. Instead, we sought to be comprehensive, included all reporting items, and did not eliminate any items based on judgement about relevance or importance. We believe this increases the practical utility of the REPRISE checklist considering the diverse range of methods and approaches that are used for research priority setting exercises unlike other reporting guidelines which are based on study designs in one particular domain.

We acknowledge that there may be other potentially relevant items that could warrant further discussion, consideration, and evidence to support their inclusion in subsequent revisions of this framework. The items addressing diversity and hierarchies amongst group members and the networks they represent, the criteria and degree of formality in decision making processes, and the medium of communication for sharing information and making decisions are all factors affecting good group decision making [53].. Additional factors, not addressed by the priority setting literature, are the size of a group making decisions, the time available for them to explore their knowledge to make choices or solve problems and the facilitation skills for managing constructive conflict. We seek further feedback from researchers, end-users and other stakeholders, to inform future efforts to refine and revise the guideline as needed.

## Conclusions

The REPRISE guideline has the potential to improve transparency in reporting research priority setting studies. Improved explicitness in how research priority setting studies are conducted could strengthen legitimacy, confidence, and acceptability of the findings, and thereby support the implementation and impact of these efforts.

## Supplementary information

**Additional file 1.** Search strategies.

**Additional file 2.** Search results.

**Additional file 3.** Research priority setting: frameworks and reviews.

**Additional file 4.** Preliminary REPRISE guideline.

**Additional file 5.** Sources contributing to the reporting items.

**Additional file 6.** Results of the pilot test.

## Data Availability

Not applicable. All guidelines and publications used for this article are available in the public domain.

## Abbreviations

CHNRI: Child Health and Nutrition Research Initiative
CINAHL: Cumulative Index for Nursing and Allied Health Literature
COHRED: Council on Health Research for Development
ENHR: Essential National Health Research
EQUATOR: Enhancing the Quality and Transparency of Health Research
JLA: James Lind Alliance
PCORI: Patient-centered outcomes research institute
REPRISE: Reporting guideline for priority setting of health research
WHO: World Health Organization
