# Supplementary material for: Reporting guideline for priority setting of health research (REPRISE)
Source: BMC Med Res Methodol. 2019 Dec 28;19:243. doi: 10.1186/s12874-019-0889-3 (PMC6935471; doi:10.1186/s12874-019-0889-3)
Supplement: Supplementary file 1 — Additional file 1. Search strategies. [file 12874_2019_889_MOESM1_ESM.docx]

**Additional File 1. Search strategies**

**MEDLINE 23^rd^ July 2019**

| \| 1 \| (research adj3 priorit$).tw. \| \| --- \| --- \| \| 2 \| (research adj3 agenda$).tw. \| \| 3 \| or/1-2 \| \| 4 \| review$.tw. \| \| 5 \| principle$.tw. \| \| 6 \| appraisal$.tw. \| \| 7 \| (guideline$ or framework$ or checklist$ or criteri$).tw. \| \| 8 \| evaluat$.tw. \| \| 9 \| concept$.tw. \| \| 10 \| reporting$.tw. \| \| 11 \| or/4-10 \| \| 12 \| 3 and 11 \| |  |  |
| --- | --- | --- | --- | --- | --- | --- | --- | --- | --- | --- | --- | --- | --- | --- | --- | --- | --- | --- | --- | --- | --- | --- | --- | --- | --- | --- |

**Embase 1980 - 23^rd^ July 2019**

| 1 | (research adj3 priorit$).tw. |
| --- | --- |
| 2 | (research adj3 agenda$).tw. |
| 3 | or/1-2 |
| 4 | review$.tw. |
| 5 | principle$.tw. |
| 6 | appraisal$.tw. |
| 7 | (guideline$ or framework$ or checklist$ or criteri$).tw. |
| 8 | evaluat$.tw. |
| 9 | concept$.tw. |
| 10 | reporting$.tw. |
| 11 | or/4-10 |
| 12 | 3 and 11 |

**PsycINFO 1806 to 23^rd^ July 2019**

| 1 | (research adj3 priorit$).tw. |
| --- | --- |
| 2 | (research adj3 agenda$).tw. |
| 3 | or/1-2 |
| 4 | review$.tw. |
| 5 | principle$.tw. |
| 6 | appraisal$.tw. |
| 7 | (guideline$ or framework$ or checklist$ or criteri$).tw. |
| 8 | evaluat$.tw. |
| 9 | concept$.tw. |
| 10 | reporting$.tw. |
| 11 | or/4-10 |
| 12 | 3 and 11 |

**CINAHL to 23^rd^ July 2019**

|  | S1 | (MH "Research Priorities") |  |
| --- | --- | --- | --- |
