# Supplementary material for: Reporting guideline for priority setting of health research (REPRISE)
Source: BMC Med Res Methodol. 2019 Dec 28;19:243. doi: 10.1186/s12874-019-0889-3 (PMC6935471; doi:10.1186/s12874-019-0889-3)
Supplement: Supplementary file 3 — Additional file 3. Research priority setting: frameworks and reviews. [file 12874_2019_889_MOESM3_ESM.docx]

**Additional File 3. Research priority setting: frameworks and reviews**

| **ID** | **Summary** |
| --- | --- |
| **Framework and guidelines for conducting or evaluating research priority setting** | |
| JLA Handbook 2018 (JLA)[[5](#_ENREF_5)] | James Lind Alliance priority setting partnership |
| Montorzi 2010 (COHRED)[[19](#_ENREF_19)] | A management process for priority setting http://wMww.cohred.org/downloads/Priority_Setting_COHRED_approach_August_2010.pdf |
| Okello 2000 (ENHR)[[39](#_ENREF_39)] | A manual for research priority setting using the ENHR strategy http://www.cohred.org/publications/library-and-archive/a_manual_for_researc_1_0/ |
| Ranson 2009[[40](#_ENREF_40)] | Health policy and systems research |
| Rudan 2008 (CHNRI)[[41](#_ENREF_41)] | Child Health and Nutrition Research Initiative (CHNRI) |
| Viergever 2010 (WHO)[[13](#_ENREF_13)] | Health research prioritisation at WHO www.who.int/rpc/publications/Health_research_prioritization_at_WHO.pdf |
| Lomas 2003[[38](#_ENREF_38)] | Setting priorities for applied health services research |
| Nasser 2012[[20](#_ENREF_20)] | Evaluating methods for prioritising topics for Cochrane reviews; also with reference to Sibbald 2009. |
| PCORI 2012[[28](#_ENREF_28)] | Executive summary of a workshop for setting research priorities |
| Abma 2012 (Dialogue Model)[[36](#_ENREF_36)] | Dialogue Model - Methodology for patient participation in research agenda setting projects |
| Dubois 2011[[37](#_ENREF_37)] | Framework for setting priorities for comparative effectiveness research |
| VanLare 2010 (IOM)[[42](#_ENREF_42)] | Five steps for a national program for comparative effectiveness research (IOM) |
| Sibbald 2009[[18](#_ENREF_18)] | A conceptual framework for successful priority setting |
| **Systematic reviews of priorities setting projects** | |
| Badakhshan 2018[[21](#_ENREF_21)] | Review of RSP conducted in the Islamic republic of Iran |
| Chanda-Kapata 2016[[22](#_ENREF_22)] | Review of health research priority setting in Zambia |
| McGregor 2014*[[43](#_ENREF_43)] | Low and middle income countries (Appraisal: used Viergever 2010) |
| Odgers 2018*[[23](#_ENREF_23)] | Systematic review of RSP in childhood chronic conditions |
| Oliver 2006*[[15](#_ENREF_15)] | Bibliography of reports http://www.jla.nihr.ac.uk/news-and-publications/downloads/Annual%20Report%202007-08/Annexe-16-2007-2008-A-Bibliography.pdf (No appraisal) |
| Reveiz 2013*[[32](#_ENREF_32)] | Comparison of national health research priority-setting methods and characteristics in Latin America and the Caribbean (Appraisal: used Viergever 2010) |
| Rylance 2010[[24](#_ENREF_24)] | Systematic review of priority setting in tuberculosis |
| Stewart 2010*[[16](#_ENREF_16)] | Patients’ and clinicians’ research priorities (No appraisal) |
| Swingler 2005[[44](#_ENREF_44)] | Review of child health research priorities in Sub-Saharan Africa |
| Terry 2018[[27](#_ENREF_27)] | Review and analysis of research priority-setting at WHO |
| Tomlinson 2011[[17](#_ENREF_17)] | Review of selected research priority setting process in local and middle-income countries |
| Tong 2015*[[25](#_ENREF_25)] | Systematic review of RPS in kidney disease (Appraisal: used Viergever 2010) |
| Tong 2017*[[26](#_ENREF_26)] | Systematic review of RPS in organ transplantation (Appraisal: used preliminary version of REPRISE) |

*not included in the candidate list as they used an existing framework, or did not conduct any appraisal.
