# Supplementary material for: Reporting guideline for priority setting of health research (REPRISE)
Source: BMC Med Res Methodol. 2019 Dec 28;19:243. doi: 10.1186/s12874-019-0889-3 (PMC6935471; doi:10.1186/s12874-019-0889-3)
Supplement: Supplementary file 4 — Additional file 4. Preliminary REPRISE guideline. [file 12874_2019_889_MOESM4_ESM.docx]

**Additional File 4. Preliminary REPRISE guideline**

| **No** | **Item** | **Descriptor and/or *examples*** |
| --- | --- | --- |
| **A** | **Context and scope** |  |
| 1 | Define geographical scope | Global, regional, national, city, local area, institutional/organisational level, health service |
| 2 | Define health area, field, focus | Disease or condition specific, interventions, healthcare delivery, health system |
| 3 | Define end-users of research | Intended beneficiaries e.g. general population or a specific population based on demographic (age, gender), clinical (disease, condition), or other characteristics |
| 4 | Define the target audience of the priorities | Policy makers, funders, researchers, industry |
| 5 | Identify the broad research area | Public health, health services research, clinical research, basic science |
| 6 | Identify the type of research question | Etiology, diagnosis, prevention, treatment (interventions), prognosis, health services, psychosocial, behavioral and social science, economic evaluation, implementation |
| 7 | Define the time frame | Interim, short-term, long-term priorities, plans to revise and update |
| **B** | **Governance and team** |  |
| 8 | Describe selection of the leadership and management team | Those responsible for initiating, developing, and guiding the process for priority setting e.g. Steering Committee, Advisory Group, Technical Experts |
| 9 | Describe the characteristics of the team, and the networks they represent | Stakeholder groups, organisations or networks represented, characteristics (demographics, experience, expertise) |
| 10 | Describe any training or experience in priority setting | Consultants or advisors with experience in priority setting |
| **C** | **Framework for priority setting** |  |
| 11 | State the framework used (if any) | James Lind Alliance, COHRED, CHNRI, no framework |
| **D** | **Stakeholders or participants** |  |
| 12 | Define the inclusion criteria for stakeholders involved in priority-setting | Patients, caregivers, general community, health professionals, researchers, policy makers, non-governmental organisations, government, industry; specific groups including vulnerable and marginalized populations |
| 13 | State the strategy or method for identifying and engaging stakeholders | Partnership with organizations, social media, recruitment through hospitals |
| 14 | Indicate the number of participants and/or organisations involved | Number of individuals and organisations, include number by stakeholder group |
| 15 | Describe the characteristics of stakeholders | Stakeholder group, demographic characteristics, areas of interest and expertise, discipline, affiliations |
| 16 | State if reimbursement for participation was provided | Cash, vouchers, certificates, acknowledgement; what purpose e.g. travel, accommodation, honorarium |
| **E** | **Identification and collection of research priorities** |  |
| 17 | Describe methods for collecting priorities from stakeholders | Methods e.g. Delphi survey, surveys, nominal group technique, interviews, focus groups, meetings, workshops; prioritization e.g. voting, ranking; mode e.g. face-to-face, online; may be informed by evidence e.g, systematic reviews, reviews of guidelines/other documents |
| 18 | Describe methods for collating and categorizing priorities | Taxonomy or other framework used to organise, summarise, and aggregate topics or questions |
| 19 | Describe methods and reasons for removing priorities | Based on scope, clarity, definition, duplication, other criteria |
| 20 | Describe methods for refining or translating priorities into research topics or questions | Reviewed by Steering Committee or project team |
| 21 | Describe methods for checking whether research questions or topics have been answered | Systematic reviews, evidence mapping, consultation with experts |
| 22 | Describe number of research questions or topics | Number of priorities at each stage of the process |
| **F** | **Prioritisation of research topics/questions** |  |
| 23 | Describe methods and criteria for prioritising research topics or questions | Methods e.g. Delphi survey, surveys, nominal group technique, interviews, focus groups, meetings, workshops; prioritisation e.g. voting, ranking; mode e.g. face-to-face, online; criteria e.g. need, feasibility, novelty, equity |
| 24 | Provide reasons for excluding research topics/questions | Thresholds for ranking scores, proportions, votes; other criteria |
| **G** | **Output** |  |
| 25 | Specificity of research priorities are clear | Area, topic, questions, PICO (population, intervention, comparator, outcome) |
| **H** | **Evaluation and feedback** |  |
| 26 | Describe how the process of prioritization was evaluated | Survey, workshop |
| 27 | Describe the approach for feeding back priorities to stakeholders and/or to the public; and how feedback was addressed and integrated | Public meetings or workshop, newsletters, website, email |
| **I** | **Implementation** |  |
| 28 | Outline the strategy or action plans for implementing priorities | Communication with target audience, via policies and funding |
| 29 | Describe evaluation of impact | Integration in decision-making, funding allocation |
| **J** | **Funding and conflict of interest** |  |
| 30 | State sources of funding | Name sources of funding for the priority-setting exercise |
| 31 | Outline the budget and/or cost | Indicate budget and cost |
| 32 | Provide declaration of conflict of interest | Statement of conflict of interest |
