# Supplementary material for: Reporting guideline for priority setting of health research (REPRISE)
Source: BMC Med Res Methodol. 2019 Dec 28;19:243. doi: 10.1186/s12874-019-0889-3 (PMC6935471; doi:10.1186/s12874-019-0889-3)
Supplement: Supplementary file 5 — Additional file 5. Sources contributing to the reporting items. [file 12874_2019_889_MOESM5_ESM.docx]

**Additional File 5. Sources contributing to the reporting items**

| **No** | **Item** | **Sources** | **Examples of original statements** | **N** |
| --- | --- | --- | --- | --- |
| **A** | **Context and scope** |  |  |  |
| 1 | Define geographical scope | [[13](#_ENREF_13), [19](#_ENREF_19), [21](#_ENREF_21), [39-41](#_ENREF_39)] | Level: they may aim to set research priorities at global, national, sub-national or institutional levels.[[40](#_ENREF_40)] | 6 |
| 2 | Define health area, field, focus | [[5](#_ENREF_5), [13](#_ENREF_13), [19-21](#_ENREF_19), [27](#_ENREF_27), [37](#_ENREF_37), [38](#_ENREF_38), [40](#_ENREF_40), [41](#_ENREF_41), [44](#_ENREF_44)] | …or the breadth of the condition or health area and its unique issues[[5](#_ENREF_5)] | 13 |
| 3 | Define end-users of research | [[5](#_ENREF_5), [13](#_ENREF_13), [20](#_ENREF_20), [21](#_ENREF_21), [37-39](#_ENREF_37), [41](#_ENREF_41), [44](#_ENREF_44)] | The target audience of the research priorities[[13](#_ENREF_13)] | 9 |
| 4 | Define the target audience of the priorities | [[13](#_ENREF_13), [17](#_ENREF_17), [39-41](#_ENREF_39)] | People affected by the research results[[39](#_ENREF_39)] | 5 |
| 5 | Identify the broad research area | [[20](#_ENREF_20), [37](#_ENREF_37), [41](#_ENREF_41), [44](#_ENREF_44)] | Basic, clinical, and public health research[[41](#_ENREF_41)] | 4 |
| 6 | Identify the type of research question | [[5](#_ENREF_5), [20](#_ENREF_20), [27](#_ENREF_27), [38](#_ENREF_38), [39](#_ENREF_39)] | …type of research questions varied widely[[24](#_ENREF_24)] | 5 |
| 7 | Define the time frame | [[13](#_ENREF_13), [17](#_ENREF_17), [19](#_ENREF_19), [37-39](#_ENREF_37), [41](#_ENREF_41), [42](#_ENREF_42), [47](#_ENREF_47)] | The intended timeframe (long-term or short-term priorities)[[13](#_ENREF_13)] | 8 |
| **B** | **Governance and team** |  |  |  |
| 8 | Describe selection of the leadership and management team | [[5](#_ENREF_5), [13](#_ENREF_13), [17](#_ENREF_17), [19-21](#_ENREF_19), [27](#_ENREF_27), [39](#_ENREF_39), [41](#_ENREF_41)] | Selecting the managers of the process[[41](#_ENREF_41)] | 9 |
| 9 | Describe the characteristics of the team | [[5](#_ENREF_5), [17](#_ENREF_17), [20](#_ENREF_20), [21](#_ENREF_21), [27](#_ENREF_27), [41](#_ENREF_41)] | How members of the committee were chosen, or who they represented[[17](#_ENREF_17)] | 6 |
| 10 | Describe any training or experience in priority setting | [[5](#_ENREF_5), [13](#_ENREF_13), [40](#_ENREF_40)] | Consulting individuals or organizations with previous experience in health research priority setting as part of the preparatory work can aid in obtaining a higher quality process for setting priorities.[[13](#_ENREF_13)] | 3 |
| **C** | **Framework for priority setting** |  |  |  |
| 11 | Indicate use of frameworks | [[22](#_ENREF_22), [27](#_ENREF_27), [28](#_ENREF_28), [44](#_ENREF_44)] | Guiding framework/ approaches employed[[22](#_ENREF_22)] | 4 |
| **D** | **Inclusion of stakeholders/participants** |  |  |  |
| 12 | Define the inclusion criteria for stakeholder groups involved in priority-setting | [[5](#_ENREF_5), [13](#_ENREF_13), [17](#_ENREF_17), [18](#_ENREF_18), [20-22](#_ENREF_20), [36-42](#_ENREF_36), [44](#_ENREF_44)] | Mix of stakeholders depends on the particular function of the research priority-setting exercise[[38](#_ENREF_38)]  Who are the main groups in the society whose values and interests should be respected in setting health research investment priorities?[[41](#_ENREF_41)] | 15 |
| 13 | State the strategy or method for identifying and engaging stakeholders | [[5](#_ENREF_5), [13](#_ENREF_13), [17](#_ENREF_17), [18](#_ENREF_18), [20](#_ENREF_20), [36-39](#_ENREF_36)] | It is essential to cooperate with people or institutions like NGOs and government bodies that have already established particularly good contacts with the community.[[39](#_ENREF_39)] | 9 |
| 14 | Indicate the number of participants and/or organisations involved | [[5](#_ENREF_5), [13](#_ENREF_13), [17](#_ENREF_17), [22](#_ENREF_22), [38](#_ENREF_38), [39](#_ENREF_39)] | How many people/groups were involved?  [[39](#_ENREF_39)] | 6 |
| 15 | Describe the characteristics of stakeholders | [[5](#_ENREF_5), [13](#_ENREF_13), [20](#_ENREF_20), [21](#_ENREF_21), [36](#_ENREF_36), [37](#_ENREF_37), [39](#_ENREF_39), [41](#_ENREF_41), [42](#_ENREF_42), [44](#_ENREF_44)] | It is important to pay attention to diversity (sex, age, SES, ethnicity)[[36](#_ENREF_36)]  Is there appropriate representation of expertises and balanced gender and regional participation?[[42](#_ENREF_42)] | 10 |
| 16 | State if reimbursement for participation was provided | [[5](#_ENREF_5)] | PSPs will need to consider reimbursement of travel expenses, accommodation if needed, payment for the time of patients/carers.[[5](#_ENREF_5)] | 1 |
| **D** | **Identification and collection of research priorities** |  |  |  |
| 17 | Describe methods for collecting priorities | [[5](#_ENREF_5), [13](#_ENREF_13), [18-22](#_ENREF_18), [27](#_ENREF_27), [28](#_ENREF_28), [36-41](#_ENREF_36)] | Method of question identification described.[[24](#_ENREF_24)]  Approaches for Topic Generation.[[28](#_ENREF_28)] | 15 |
| 18 | Describe methods for collating/categorising priorities | [[5](#_ENREF_5), [18](#_ENREF_18), [36](#_ENREF_36), [39-41](#_ENREF_39)] | Participants had an opportunity to take an active part in compiling the list of priority topics.[[22](#_ENREF_22)] | 6 |
| 19 | Describe methods and reasons for removing priorities | [[5](#_ENREF_5), [39-41](#_ENREF_39)] | Remove out-of-scope survey submissions.[[5](#_ENREF_5)] | 4 |
| 20 | Describe methods for refining or translating priorities into research topics/questions | [[5](#_ENREF_5), [22](#_ENREF_22), [28](#_ENREF_28), [38](#_ENREF_38), [39](#_ENREF_39), [41](#_ENREF_41)] | A team of experts synthesised the input from stakeholders.[[22](#_ENREF_22)]  The clarifying statement should be written in a format that can be quantitatively scored at the next stage.[[39](#_ENREF_39)] | 6 |
| 21 | Describe methods for checking whether research questions/topics have been answered | [[5](#_ENREF_5), [39](#_ENREF_39)] | Using the literature to check whether survey-submitted uncertainties have already been answered or not.[[5](#_ENREF_5)] | 2 |
| 22 | State number of research questions/topics | [[5](#_ENREF_5), [20](#_ENREF_20), [37](#_ENREF_37), [38](#_ENREF_38), [40](#_ENREF_40)] | The number and type of research questions varied widely across the articles.[[24](#_ENREF_24)] | 5 |
| **E** | **Prioritisation of research topics/questions** |  |  |  |
| 23 | Describe methods and criteria for prioritizing research areas/topics/questions | [[5](#_ENREF_5), [13](#_ENREF_13), [17-22](#_ENREF_17), [27](#_ENREF_27), [36-42](#_ENREF_36)] | Valid criteria are used with complete explanation about score points and scoring systems identification. [[21](#_ENREF_21)]  Decide on technique to be used to rank priority issues.[[19](#_ENREF_19)]  Method of prioritisation described.[[24](#_ENREF_24)]  Choose a method for deciding on priorities. Decide whether to use a consensus based approach or a metrics based approach (pooling individual rankings), or a combination.[[13](#_ENREF_13)] | 16 |
| 24 | Provide reasons for excluding research topics/questions | [[5](#_ENREF_5), [18](#_ENREF_18)] | Remove out-of-scope survey submissions.[[5](#_ENREF_5)] | 2 |
| **F** | **Output** |  |  |  |
| 25 | Specificity of research priorities are clear | [[5](#_ENREF_5), [37-39](#_ENREF_37)] | PICO is a helpful framework to aim for, but the JLA recognises that not all the PICO variables will necessarily be available.[[5](#_ENREF_5)]  Is the research agenda specific enough to guide donors and researchers?[[39](#_ENREF_39)] | 4 |
| **G** | **Evaluation and feedback** |  |  |  |
| 26 | Describe how the process of prioritisation was evaluated | [[5](#_ENREF_5), [13](#_ENREF_13), [18-20](#_ENREF_18), [37](#_ENREF_37)] | Evaluate the prioritization process.[[37](#_ENREF_37)] | 6 |
| 27 | Describe the approach for feeding back priorities to stakeholders and/or to the public; and how feedback was addressed and integrated | [[18](#_ENREF_18), [21](#_ENREF_21), [22](#_ENREF_22), [36](#_ENREF_36), [39](#_ENREF_39)] | Appeal/publicizing. Using mechanisms such as public meetings and newsletters, with a mechanism for getting feedback.[[21](#_ENREF_21)]  A revision process is a formal mechanism for reviewing decisions and for addressing disagreements constructively.[[18](#_ENREF_18)] | 5 |
| **H** | **Translation and implementation** |  |  |  |
| 28 | Outline the strategy or action plans for implementing priorities | [[5](#_ENREF_5), [13](#_ENREF_13), [19](#_ENREF_19), [21](#_ENREF_21), [22](#_ENREF_22), [36](#_ENREF_36), [38](#_ENREF_38), [39](#_ENREF_39)] | Provided recommendations about implementation.[[21](#_ENREF_21)]  The final phase in the process aims to implement the research programme.[[36](#_ENREF_36)]  Establish plans for translation of the priorities to actual research (via policies and funding) as a priority at the beginning of the process.[[13](#_ENREF_13)] | 8 |
| 29 | Describe evaluation of impact | [[5](#_ENREF_5), [13](#_ENREF_13), [18](#_ENREF_18), [19](#_ENREF_19), [22](#_ENREF_22), [37-39](#_ENREF_37)] | However, there was no evaluation of the extent to which these priorities were implemented.[[22](#_ENREF_22)]  How priorities are being integrated in the national research for health agenda.[[19](#_ENREF_19)] | 8 |
| **I** | **Funding and conflict of interest** |  |  |  |
| 30 | State sources of funding | [[13](#_ENREF_13)] | Are there any external demands for the exercise (e.g. political or commercial) that have an influence?[[13](#_ENREF_13)] | 1 |
| 31 | Outline the budget and/or cost | [[5](#_ENREF_5), [13](#_ENREF_13), [19](#_ENREF_19)] | Timelines and budget.[[19](#_ENREF_19)] | 3 |
| 32 | Provide declaration of conflict of interest | [[5](#_ENREF_5), [13](#_ENREF_13), [35](#_ENREF_35)] | A trans-parent method should be agreed upon to manage potential conflicts of interest in personal, professional and commercial areas.[[13](#_ENREF_13)] | 3 |
