# Supplementary material for: Reporting guideline for priority setting of health research (REPRISE)
Source: BMC Med Res Methodol. 2019 Dec 28;19:243. doi: 10.1186/s12874-019-0889-3 (PMC6935471; doi:10.1186/s12874-019-0889-3)
Supplement: Supplementary file 6 — Additional file 6. Results of the pilot test. [file 12874_2019_889_MOESM6_ESM.docx]

**Additional File 6. Results of the pilot test**

1. **Summary of articles included in the pilot test (using the preliminary REPRISE Guideline – see Additional File 4)**

| **ID** | **Author/year** | **Health topic/scope** | **Region** | **Stakeholder/participants** | **N** | **Framework and outline of methods** | **Output** |
| --- | --- | --- | --- | --- | --- | --- | --- |
| 1 | Abu-Rmeileh 2018[[48](#_ENREF_48)] | Reproductive health in Palestinian territory | Palestinian territory | Reproductive health experts | 30 | CHNRI; scored against 5 criteria of answerability, effectiveness, deliverability, impact, equity | 1239 collected, 50 research priority questions |
| 2 | Acosta 2019[[49](#_ENREF_49)] | Aortic dissection | Scandinavia | Patients, caregivers, clinicians | 120 | JLA; survey, workshop | 10 research uncertainties |
| 3 | Armstrong 2012[[50](#_ENREF_50)] | Psoriasis and psoriatic arthritis | International | Clinicians | NS | Meeting with panel discussion and panel discussion | Research areas |
| 4 | Arora 2017[[51](#_ENREF_51)] | Maternal and child health, and nutrition | India | Experts (researchers clniicians, policy makers, mangers, agriculturists) | 498 | CHNRI; scored against 5 criteria of answerability, relevance, equity, innovation, investment on research | 4002 research ideas, 373 research options |
| 5 | Azeredo 2014[[52](#_ENREF_52)] | Access to medicine | Latin America and Carribean | Researchers, NGO, international agencies, government, pharmaceutical companies | 84 | Interviews, surveys | 5 most relevant criteria to define research priorities |
| 6 | Biccard 2016[[53](#_ENREF_53)] | Perioperative research | South Africa | NS | NS | Delphi technique including surveys and workshop | 166 submitted, 10 research priorities |
| 7 | Dear 2012[[54](#_ENREF_54)] | Colorectal cancer | Australia | Consumers, health professionals, researchers, funders | 43 | Consensus meeting | 4 research areas |
| 8 | De Haan 2015[[55](#_ENREF_55)] | Science, technology and health | Tanzania | Universities, R&D institutions, government, private sector, civil society | 302 | EHNR, Combined Matrix Approach, Advisory Committee, Ad Hoc Committee, CHNRI; workshops; ranked based on appropriateness, relevance, feasibility, impact, partnership | 20 per sector |
| 9 | Gierisch 2014[[56](#_ENREF_56)] | Ductal carcinoma in situ | US | Clinical experts and researchers, funders, healthcare decision makers, policy makers, consumer and patient advocacy groups | 9 | Webinar, forced ranking prioritisation | 10 research areas |
| 10 | Goold 2018[[57](#_ENREF_57)] | Health research for minority and underserved communities | US | Members from minority and medically underserved communities | 519 | Surveys, simulation exercise (CHAT) | 5 research areas |
| 11 | Gordon 2017[[58](#_ENREF_58)] | Alcohol, HIV/AIDs | International (LMIC) | Experts | 171 | CHNRI; scored against criteria of answerability, effectiveness, feasibility, applicability, impact, equity | 205 research ideas, 48 questions evaluated |
| 12 | Gregorio 2012[[59](#_ENREF_59)] | Mental health | Brazil | Researchers, policy makers, coordinator | 28 | CHNRI; scored against criteria of effectiveness, feasibility, disease burden, equity | 110 collected, 35 questions |
| 13 | Hauck 2017[[60](#_ENREF_60)] | Sudden infant death | International (25 countries) | Researchers, clinicians, counsellors, educators, parents | >600 | Online surveys, 3 workshops | 10 priority areas |
| 14 | Jang 2015[[61](#_ENREF_61)] | Cancer | Korea | Experts | 34 | Delphi survey | 7 priority research plans |
| 15 | Kelly 2018[[62](#_ENREF_62)] | Respiratory nursing | UK | Members of professional respiratory organisations | 183 | Online Delphi survey | Top 5 priorities |
| 16 | Krishnan 2013[[63](#_ENREF_63)] | Chronic obstructive pulmonsry disease | US | Patient advocacy groups, private health plans/payers, physician and nonphysician professional organisations, quality improvement organisations, industry, research organisations, funders | >70 | Discussions and provisional voting by teleconference and email, workshop, modified Delphi Technique | 4 topics |
| 17 | Lambert 2019[[64](#_ENREF_64)] | Caregivers in cancer | Canada | Caregivers, clinicians, managers, researchers | 249 | Online Delphi survey | 86 topics, 10 content areas |
| 18 | Lawn 2011[[65](#_ENREF_65)] | Birth Asphyxia | International | Experts | 21 | CHNRI; scored against criteria of answerability, effectiveness, feasibility, impact, equity | Top 15 priorities |
| 19 | Lindson 2017[[66](#_ENREF_66)] | Tobacco control | International (28 countries) | Public, clinicians, researchers, funders, commissioners, public health organisations | 304 | Two surveys, workshop | 183 submitted, 15 categories, 5 themes |
| 20 | Manikam 2017[[67](#_ENREF_67)] | Health research in South Asian children | UK | Healthcare professionals, South Asian adolescents and families | 62 | Scoping survey, focus groups | Research priority areas |
| 21 | Mikton 2017[[68](#_ENREF_68)] | Interpersonal violence prevention | International | Experts | >280 | CHNRI; Delphi survey, rating against criteria of significance, feasibility, applicability, equity, ethics | 34 interventions |
| 22 | O’Neill 2018[[69](#_ENREF_69)] | Primary care | International (27 countries) | Public, health professionals, researchers, policy makers | 131 | JLA; Delphi survey, forum | 379 submitted, 10 priorities |
| 23 | Rees 2017[[70](#_ENREF_70)] | Gestational diabetes | Canada | Women with GDM and clinicians | 75 | JLA, survey/review of guidelines, workshop and nominal group technique | 389 submission, top 10 |
| 24 | Rowe 2014[[71](#_ENREF_71)] | Sight loss and vision | UK | Patients, carers, eye health professionals | 2220 | JLA, survey, workshops | 4461 submissions, top questions for each of the 12 categories |
| 25 | Sakashita 2018[[72](#_ENREF_72)] | Bereavement in cancer | Japan | Family members | 10157 | Survey | 1658 codes, 8 categories |
| 26 | Schölvinck 2019[[73](#_ENREF_73)] | Haematological cancer | The Netherlands | Patients | 224 | Dialogue Model (participatory research); interviews, focus groups, survey, stakeholder dialogue meeting | 6 themes with research topics |
| 27 | Schwatrz 2019[[74](#_ENREF_74)] | Pharmacotherapy in older adults with CVD | US | Officers in professionals societies, editors, trialists, division chiefs, NIH, FDA, CMS, academia, PCORI, AHRQ, pharmaceutical industry, trainees, early career faculty | NS | Series of workshops | Summary of recommendations |
| 28 | Tulsky 2017[[75](#_ENREF_75)] | Communication between clinicians and patients with serious illness | US | Experts | NS | Conference, nominal group technique | 5 research areas |
| 29 | Wojcieszek 2019[[76](#_ENREF_76)] | Pregnancy and still birth | International | Multidisciplinary experts | 79 | Online survey; GRADE rating based on importance | 16 candidate research topics for four interventions; 5 priorities |
| 30 | Zimmerman 2017[[77](#_ENREF_77)] | Diabetes and hypertension | US | Patients, public, clinicians, health educators | NS | Interviews, focus groups, voting | 1. uestions prioritised |

1. **Pilot results**

|  |  | **Study ID** | | | | | | | | | |
| --- | --- | --- | --- | --- | --- | --- | --- | --- | --- | --- | --- |
| **No** | **Item** | **1** | **2** | **3** | **4** | **5** | **6** | **7** | **8** | **9** | **10** |
| **A** | **Context and scope** |  |  |  |  |  |  |  |  |  |  |
| 1 | Define geographical scope | Y | Y | N | Y | Y | Y | Y | Y | Y | N |
| 2 | Define health area, field, focus | Y | Y | Y | Y | Y | Y | N | Y | Y | Y |
| 3 | Define end-users of research | N | Y | Y | Y | N | N | N | Y | Y | N |
| 4 | Define the target audience of the priorities | Y | Y | N | Y | N | Y | Y | N | Y | Y |
| 5 | Identify the broad research area | Y | N | N | Y | Y | Y | N | Y | Y | N |
| 6 | Identify the type of research question | Y | N | N | Y | N | N | N | Y | Y | N |
| 7 | Define the time frame | N | N | N | Y | N | Y | Y | N | N | N |
| **B** | **Governance and team** |  |  |  |  |  |  |  |  |  |  |
| 8 | Describe selection the leadership and management team | N | Y | N | N | N | N | Y | Y | Y | Y |
| 9 | Describe the characteristics of the team | N | Y | N | Y | N | N | N | N | N | Y |
| 10 | Describe any training or experience in priority setting | N | N | N | N | N | N | Y | N | N | N |
| **C** | **Framework for priority setting** |  |  |  |  |  |  |  |  |  |  |
| 11 | State the framework used (if any) | Y | Y | N | Y | Y | N | N | N | N | Y |
| **D** | **Inclusion of stakeholders/participants** |  |  |  |  |  |  |  |  |  |  |
| 12 | Define the inclusion criteria for stakeholders involved in priority-setting | Y | Y | N | Y | Y | N | Y | Y | Y | Y |
| 13 | State the strategy or method for identifying and engaging stakeholders | Y | Y | N | Y | Y | Y | Y | N | Y | Y |
| 14 | Indicate the number of participants and/or organisations involved | Y | Y | N | Y | Y | N | Y | Y | Y | Y |
| 15 | Describe the characteristics of stakeholders | Y | Y | N | Y | Y | N | Y | N | N | Y |
| 16 | State if reimbursement for participation was provided | N | N | N | N | N | N | N | N | N | N |
| **D** | **Identification and collection of research priorities** |  |  |  |  |  |  |  |  |  |  |
| 17 | Describe methods for collecting priorities from stakeholders | Y | Y | Y | Y | Y | Y | Y | Y | Y | Y |
| 18 | Describe methods for collating and categorising priorities | Y | Y | N | Y | Y | Y | N | Y | Y | Y |
| 19 | Describe methods and reasons for removing priorities | N | Y | N | Y | N | N | Y | N | Y | Y |
| 20 | Describe methods for refining or translating priorities into research topics or questions | Y | ? | N | Y | N | N | N | Y | Y | N |
| 21 | Describe methods for checking whether research questions or topics have been answered | N | N | N | N | Y | N | Y | Y | Y | N |
| 22 | Describe number of research questions or topics | Y | Y | Y | Y | Y | Y | Y | N | Y | Y |
| **E** | **Prioritisation of research topics/questions** |  |  |  |  |  |  |  |  |  |  |
| 23 | Describe methods and criteria for prioritising research topics or questions | Y | Y | N | Y | Y | Y | Y | Y | Y | Y |
| 24 | Provide reasons for excluding research topics/questions | N | Y | N | Y | N | Y | Y | Y | Y | N |
| **F** | **Output** |  |  |  |  |  |  |  |  |  |  |
| 25 | Specificity of research priorities are clear | Y | Y | Y | N | N | Y | Y | Y | Y | Y |
| **G** | **Evaluation and feedback** |  |  |  |  |  |  |  |  |  |  |
| 26 | Describe how the process of prioritisation was evaluated | N | N | N | N | N | N | N | N | N | N |
| 27 | Describe the approach for feeding back priorities to stakeholders and/or to the public; and how feedback was addressed and integrated | N | N | N | N | N | N | N | N | N | N |
| **H** | **Translation and implementation** |  |  |  |  |  |  |  |  |  |  |
| 28 | Outline the strategy or action plans for implementing priorities | N | N | N | N | N | N | Y | N | Y | N |
| 29 | Describe evaluation of impact | N | N | N | N | N | N | N | N | N | N |
| **I** | **Funding and conflict of interest** |  |  |  |  |  |  |  |  |  |  |
| 30 | State sources of funding | Y | Y | N | Y | Y | Y | N | Y | Y | Y |
| 31 | Outline the budget and/or cost | N | N | N | N | N | N | N | N | N | N |
| 32 | Provide declaration of conflict of interest | Y | Y | N | Y | Y | N | Y | Y | Y | Y |

Y, reported on the item (or could be inferred); N, not reported

|  |  | **Study ID** | | | | | | | | | |
| --- | --- | --- | --- | --- | --- | --- | --- | --- | --- | --- | --- |
| **No** | **Item** | **11** | **12** | **13** | **14** | **15** | **16** | **17** | **18** | **19** | **20** |
| **A** | **Context and scope** |  |  |  |  |  |  |  |  |  |  |
| 1 | Define geographical scope | Y | Y | Y | Y | Y | Y | Y | Y | Y | Y |
| 2 | Define health area, field, focus | Y | Y | Y | Y | Y | Y | Y | Y | Y | Y |
| 3 | Define end-users of research | Y | N | Y | Y | Y | Y | Y | Y | Y | Y |
| 4 | Define the target audience of the priorities | N | N | Y | N | Y | Y | Y | Y | Y | Y |
| 5 | Identify the broad research area | Y | Y | Y | Y | Y | Y | Y | Y | Y | Y |
| 6 | Identify the type of research question | Y | Y | Y | Y | N | Y | Y | Y | Y | Y |
| 7 | Define the time frame | Y | Y | N | N | Y | Y | Y | Y | Y | N |
| **B** | **Governance and team** |  |  |  |  |  |  |  |  |  |  |
| 8 | Describe selection the leadership and management team | N | Y | Y | N | N | Y | Y | Y | Y | Y |
| 9 | Describe the characteristics of the team | Y | Y | Y | N | N | N | Y | Y | Y | Y |
| 10 | Describe any training or experience in priority setting | N | N | Y | N | N | N | Y | N | N | Y |
| **C** | **Framework for priority setting** |  |  |  |  |  |  |  |  |  |  |
| 11 | State the framework used (if any) | Y | Y | Y | Y | Y | N | Y | Y | Y | N |
| **D** | **Inclusion of stakeholders/participants** |  |  |  |  |  |  |  |  |  |  |
| 12 | Define the inclusion criteria for stakeholders involved in priority-setting | N | Y | Y | N | Y | Y | Y | Y | Y | Y |
| 13 | State the strategy or method for identifying and engaging stakeholders | Y | N | N | N | Y | Y | Y | Y | Y | Y |
| 14 | Indicate the number of participants and/or organisations involved | Y | Y | Y | Y | Y | Y | Y | Y | Y | Y |
| 15 | Describe the characteristics of stakeholders | N | Y | Y | N | Y | Y | Y | Y | Y | Y |
| 16 | State if reimbursement for participation was provided | N | N | N | N | N | N | N | N | Y | N |
| **D** | **Identification and collection of research priorities** |  |  |  |  |  |  |  |  |  |  |
| 17 | Describe methods for collecting priorities from stakeholders | Y | N | Y | Y | Y | Y | Y | Y | Y | Y |
| 18 | Describe methods for collating and categorising priorities | Y | N | Y | Y | Y | Y | Y | Y | Y | Y |
| 19 | Describe methods and reasons for removing priorities | Y | N | Y | N | Y | N | Y | N | Y | N |
| 20 | Describe methods for refining or translating priorities into research topics or questions | N | N | Y | N | N | Y | Y | Y | Y | Y |
| 21 | Describe methods for checking whether research questions or topics have been answered | N | N | Y | Y | Y | Y | N | Y | Y | Y |
| 22 | Describe number of research questions or topics | Y | Y | Y | Y | Y | Y | Y | Y | Y | Y |
| **E** | **Prioritisation of research topics/questions** |  |  |  |  |  |  |  |  |  |  |
| 23 | Describe methods and criteria for prioritising research topics or questions | Y | Y | Y | Y | Y | Y | Y | Y | Y | Y |
| 24 | Provide reasons for excluding research topics/questions | Y | N | N | Y | Y | N | Y | N | Y | N |
| **F** | **Output** |  |  |  |  |  |  |  |  |  |  |
| 25 | Specificity of research priorities are clear | Y | Y | Y | Y | N | Y | Y | Y | Y | Y |
| **G** | **Evaluation and feedback** |  |  |  |  |  |  |  |  |  |  |
| 26 | Describe how the process of prioritisation was evaluated | Y | N | N | Y | N | Y | N | Y | Y | Y |
| 27 | Describe the approach for feeding back priorities to stakeholders and/or to the public; and how feedback was addressed and integrated | N | N | N | N | N | Y | Y | Y | N | N |
| **H** | **Translation and implementation** |  |  |  |  |  |  |  |  |  |  |
| 28 | Outline the strategy or action plans for implementing priorities | N | N | Y | N | Y | Y | Y | Y | Y | N |
| 29 | Describe evaluation of impact | N | N | N | N | N | N | N | Y | Y | N |
| **I** | **Funding and conflict of interest** |  |  |  |  |  |  |  |  |  |  |
| 30 | State sources of funding | Y | N | Y | Y | Y | Y | Y | Y | Y | Y |
| 31 | Outline the budget and/or cost | N | N | N | N | N | N | N | N | Y | N |
| 32 | Provide declaration of conflict of interest | Y | N | Y | N | Y | Y | Y | Y | Y | Y |

Y, reported on the item (or could be inferred); N, not reported

|  |  | **Study ID** | | | | | | | | | |
| --- | --- | --- | --- | --- | --- | --- | --- | --- | --- | --- | --- |
| **No** | **Item** | **21** | **22** | **23** | **24** | **25** | **26** | **27** | **28** | **29** | **30** |
| **A** | **Context and scope** |  |  |  |  |  |  |  |  |  |  |
| 1 | Define geographical scope | Y | Y | Y | Y | Y | Y | Y | N | Y | N |
| 2 | Define health area, field, focus | Y | Y | Y | Y | Y | Y | Y | Y | Y | Y |
| 3 | Define end-users of research | Y | Y | Y | Y | Y | Y | Y | Y | Y | Y |
| 4 | Define the target audience of the priorities | Y | Y | Y | Y | Y | Y | Y | Y | Y | Y |
| 5 | Identify the broad research area | Y | Y | Y | Y | Y | Y | Y | Y | Y | Y |
| 6 | Identify the type of research question | Y | Y | Y | Y | Y | Y | Y | Y | Y | Y |
| 7 | Define the time frame | Y | Y | Y | Y | Y | Y | Y | Y | Y | N |
| **B** | **Governance and team** |  |  |  |  |  |  |  |  |  |  |
| 8 | Describe selection the leadership and management team | Y | N | Y | Y | N | Y | Y | N | N | N |
| 9 | Describe the characteristics of the team | N | N | Y | Y | N | Y | Y | N | Y | N |
| 10 | Describe any training or experience in priority setting | N | N | Y | N | N | N | N | N | N | N |
| **C** | **Framework for priority setting** |  |  |  |  |  |  |  |  |  |  |
| 11 | State the framework used (if any) | Y | Y | Y | Y | N | N | N | N | N | Y |
| **D** | **Inclusion of stakeholders/participants** |  |  |  |  |  |  |  |  |  |  |
| 12 | Define the inclusion criteria for stakeholders involved in priority-setting | N | Y | N | Y | Y | Y | Y | N | Y | Y |
| 13 | State the strategy or method for identifying and engaging stakeholders | N | Y | Y | Y | Y | Y | Y | Y | Y | Y |
| 14 | Indicate the number of participants and/or organisations involved | N | Y | Y | Y | Y | Y | Y | N | Y | Y |
| 15 | Describe the characteristics of stakeholders | Y | N | Y | Y | N | Y | N | N | Y | Y |
| 16 | State if reimbursement for participation was provided | N | N | N | N | N | N | N | N | N | Y |
| **D** | **Identification and collection of research priorities** |  |  |  |  |  |  |  |  |  |  |
| 17 | Describe methods for collecting priorities from stakeholders | Y | Y | Y | Y | Y | Y | Y | Y | Y | Y |
| 18 | Describe methods for collating and categorising priorities | Y | Y | Y | Y | Y | Y | N | Y | Y | Y |
| 19 | Describe methods and reasons for removing priorities | N | Y | Y | Y | N | N | N | N | N | N |
| 20 | Describe methods for refining or translating priorities into research topics or questions | Y | Y | Y | Y | N | Y | N | Y | Y | Y |
| 21 | Describe methods for checking whether research questions or topics have been answered | N | Y | Y | Y | N | Y | N | N | Y | Y |
| 22 | Describe number of research questions or topics | Y | Y | Y | Y | Y | Y | Y | Y | Y | Y |
| **E** | **Prioritisation of research topics/questions** |  |  |  |  |  |  |  |  |  |  |
| 23 | Describe methods and criteria for prioritising research topics or questions | Y | Y | Y | Y | Y | Y | N | Y | Y | Y |
| 24 | Provide reasons for excluding research topics/questions | N | Y | Y | Y | N | Y | N | N | N | N |
| **F** | **Output** |  |  |  |  |  |  |  |  |  |  |
| 25 | Specificity of research priorities are clear | Y | Y | Y | Y | Y | Y | Y | Y | Y | Y |
| **G** | **Evaluation and feedback** |  |  |  |  |  |  |  |  |  |  |
| 26 | Describe how the process of prioritisation was evaluated | Y | Y | Y | Y | Y | Y | N | N | Y | Y |
| 27 | Describe the approach for feeding back priorities to stakeholders and/or to the public; and how feedback was addressed and integrated | N | N | N | N | N | Y | N | Y | Y | Y |
| **H** | **Translation and implementation** |  |  |  |  |  |  |  |  |  |  |
| 28 | Outline the strategy or action plans for implementing priorities | N | N | Y | Y | N | Y | N | N | N | Y |
| 29 | Describe evaluation of impact | N | N | Y | N | N | N | N | N | N | Y |
| **I** | **Funding and conflict of interest** |  |  |  |  |  |  |  |  |  |  |
| 30 | State sources of funding | y | Y | Y | Y | Y | Y | Y | Y | Y | Y |
| 31 | Outline the budget and/or cost | n | N | N | N | N | N | N | N | N | N |
| 32 | Provide declaration of conflict of interest | y | Y | N | Y | Y | Y | Y | Y | Y | Y |

Y, reported on the item (or could be inferred); N, not reported
